# Thrombectomy for delayed thromboembolism in a recurrent cerebral aneurysm previously treated with coiling: A case report

**DOI:** 10.1097/MD.0000000000037403

**Published:** 2024-03-22

**Authors:** Ya Shao, Yuan Yang, Haidong Huang, Ting Wang, Jinglun Li, Yushan Jiang, Ziwei Yuan, Jiayi Tang, Dihu Wang, Zerui Xiang, Xue Zeng, Zhou Yu, Zhongchun He, Zhengzhou Yuan

**Affiliations:** a Department of Neurology, Gansu Provincial Hospital of Traditional Chinese Medicine, Lanzhou, China; b Department of Neurology, The Affiliated Hospital of Southwest Medical University, Luzhou, China; c Department of Neurosurgery, The General Hospital of Western Theater Command, Chengdu, China; d Department of Neurosurgery, West China Hospital, Sichuan University, Chengdu, China; e School of Clinical Medicine, Southwest Medical University, Luzhou, China; f Department of Neurology, The First People’s Hospital of Liangshan Yi Autonomous Prefecture, Liangshan, China; g Department of Neurology, The First Affiliated Hospital of Chengdu Medical College, Chengdu, China.

**Keywords:** case report, cerebral aneurysm, clipping, coiling, thrombectomy

## Abstract

**Rationale:**

Giant intracranial aneurysms pose a significant threat due to high mortality rates upon rupture, prompting interventions such as neurosurgical clipping or endovascular coiling.

**Patient Concerns:**

We present a rare case involving a 47-year-old female with a history of successfully treated ruptured giant intracranial aneurysms. Six months post-surgical clipping, she developed symptoms of acute ischemic stroke, prompting the decision for neurosurgical coiling and stent-assisted aneurysm coil embolization due to recurrent intracranial aneurysms.

**Diagnoses:**

Subsequently, occlusion occurred at the previously implanted stent site during embolization, necessitating exploration of alternative therapeutic options. Digital subtraction angiography confirmed stent occlusion in the right middle cerebral artery.

**Interventions:**

Despite an initial unsuccessful attempt using a direct aspiration first-pass technique, the patient underwent successful mechanical thrombectomy with a retrievable stent, leading to successful reperfusion. This study aims to highlight the challenges and therapeutic strategies in managing delayed cerebral vascular occlusion following stent-assisted coil embolization, emphasizing the significance of exploring alternative interventions to enhance patient outcomes.

**Outcomes:**

The patient achieved successful reperfusion, and the study underscores the importance of recognizing and addressing delayed cerebral vascular occlusion after stent-assisted coil embolization for recurrent cerebral aneurysms.

**Lessons:**

Our findings suggest that retrievable stent mechanical thrombectomy may serve as a viable therapeutic option in challenging scenarios, emphasizing the need for further exploration of alternative interventions to enhance patient care.

## 1. Introduction

Giant intracranial aneurysms present a significant risk of rupture, with mortality rates ranging from 65% to 100% within 1 to 5 years following rupture.^[1,2]^ While both neurosurgical clipping and endovascular coiling have demonstrated effectiveness in treatment,^[3]^ the choice between them is influenced by certain factors. For ruptured cerebral aneurysms, microsurgical clipping is often preferred due to limitations associated with antiplatelet agents.^[4]^ In comparison, patients who undergo clipping tend to achieve higher rates of total occlusion than those who opt for coiling.^[5]^ However, it is worth noting that there is a risk of recurrent intracranial aneurysms (RAs) after clipping, with an occurrence rate of 5% to 9%.^[6]^ The management of RAs following clipping poses significant challenges, prompting endovascular coiling to become the primary treatment option. Notably, thromboembolic events, though relatively rare (2%–4.9%), predominantly occur within the initial 72 hours post-procedure, with delayed embolism incidents accounting for <1% of cases.^[7]^ We present a unique case in which delayed thromboembolism was observed in a previously coiled recurrent aneurysm. This uncommon scenario was successfully managed using mechanical thrombectomy (MT) with the application of a retrievable stent.

## 2. Case report

**Figure 1.**
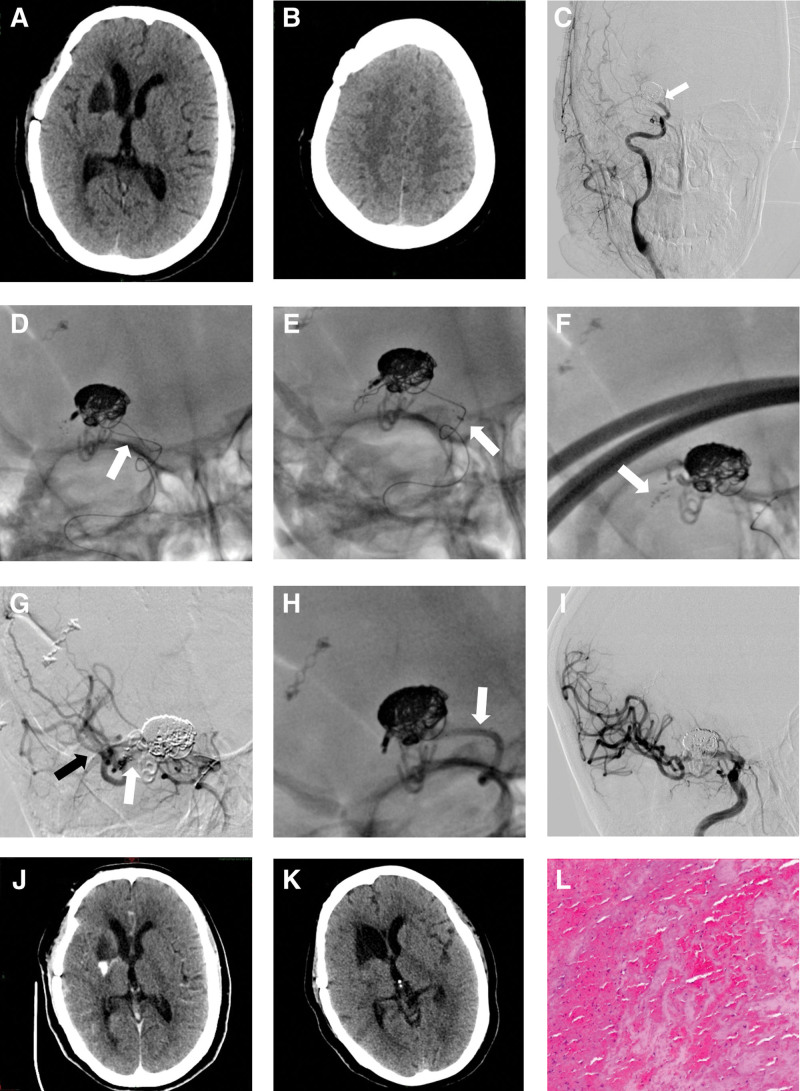
Mechanical embolectomy process of the right middle cerebral artery. (A–B) The Alberta Stroke Program Early CT Score (ASPECTS) on non-contrast CT scan was 9 points upon admission. (C) DSA indicates occlusion of the right middle cerebral artery, which had previously been treated with an implanted stent to assist with embolic coiling. (D) A guidewire loop through the occlusion of the middle cerebral artery to the distal of the stent. (E) Despite repeated attempts, the suction catheter (white arrow) was unable to reach the occlusion. (F) A Solitaire FR retrievable stent was deployed (white arrow pointing to the released stent mark point). (G) Angiography showed the retrievable stent with a clot (white arrow) and the distal branch of the middle cerebral artery (black arrow). (H) Withdraw the retrievable stent (white arrow pointing to the mark for Solitaire FR stent). (I) Successful recanalization was achieved after thrombectomy. (J–K) Non-contrast brain CT obtained immediately (J) and 24 hours (K) post-thrombectomy. (L) Cerebral clot in hematoxylin-eosin (HE) staining (100X). CT = computed tomography.

A 47-year-old female patient with a history of treated ruptured intracranial aneurysms presented with sudden-onset left-sided hemiparesis, dysarthria, and partial gaze palsy. She arrived at our hospital within 3 hours of the last known well time, and her baseline National Institutes of Health Stroke Scale score was 17. Her modified Rankin Scale score (mRS) before the stroke was 0, and her blood pressure was within the normal range, with a body mass index of 29.3. Two years prior, the patient had undergone neurosurgical clipping for a subarachnoid hemorrhage caused by a ruptured large middle cerebral artery aneurysm. Six months ago, she underwent stent-assisted coil embolization of the recurrent aneurysm in another hospital. Emergency non-contrast computed tomography (CT) scans at our hospital ruled out cerebral hemorrhage or hypoattenuation of the right cerebral hemisphere, with an Alberta Stroke Program Early CT Score of 9 points (Fig. 1A and B).

Considering the patient symptoms, medical history, and prior subarachnoid hemorrhage, we refrained from administering intravenous thrombolytic therapy after admission. Digital subtraction angiography revealed occlusion of the right middle cerebral artery, attributed to the stent implanted during the previous aneurysm embolization (Fig. 1C). Subsequently, MT was performed after obtaining informed consent from the patient husband.

We inserted a 6F Neuron Max sheath (Penumbra Inc., Alameda, CA, USA) into the right internal carotid artery. The Penumbra ACE 68 aspiration catheter (Penumbra Inc.) was advanced to the distal ICA segment using the Velocity microcatheter (Penumbra Inc.) and Synchro-14 guidewire (Stryker, Kalamazoo, MI) (Fig. 1D). The aneurysm clip and coil adjacent to the occluded middle cerebral artery. While attempting to advance the aspiration catheter to the proximal end of the occluded vessel, several attempts were unsuccessful (Fig. 1E). To address this issue, we deployed the Solitaire FR 6 mm × 30 mm stent retriever (Medtronic, Irvine, CA) into the right middle cerebral artery (MCA) (Fig. 1F), and the aspiration catheter was successfully advanced to the MCA (Fig. 1G). A contrast injection confirmed the thrombus distal location in the right MCA. Several attempts were made to extract the stent retriever under continuous aspiration from the aspiration catheter. However, extraction was complicated by the entanglement of the Solitaire FR and the aneurysm embolization-assisted stent. Eventually, the stent retriever was successfully withdrawn after multiple attempts (Fig. 1H), but the MCA remained occluded. Successful reperfusion was achieved with a total of 3 passes (Fig. 1I). Post-thrombectomy, a non-contrast head CT was performed (Fig. 1J–K), and the retrieved clot was examined pathologically (Fig. 1L).

**Figure 2.**
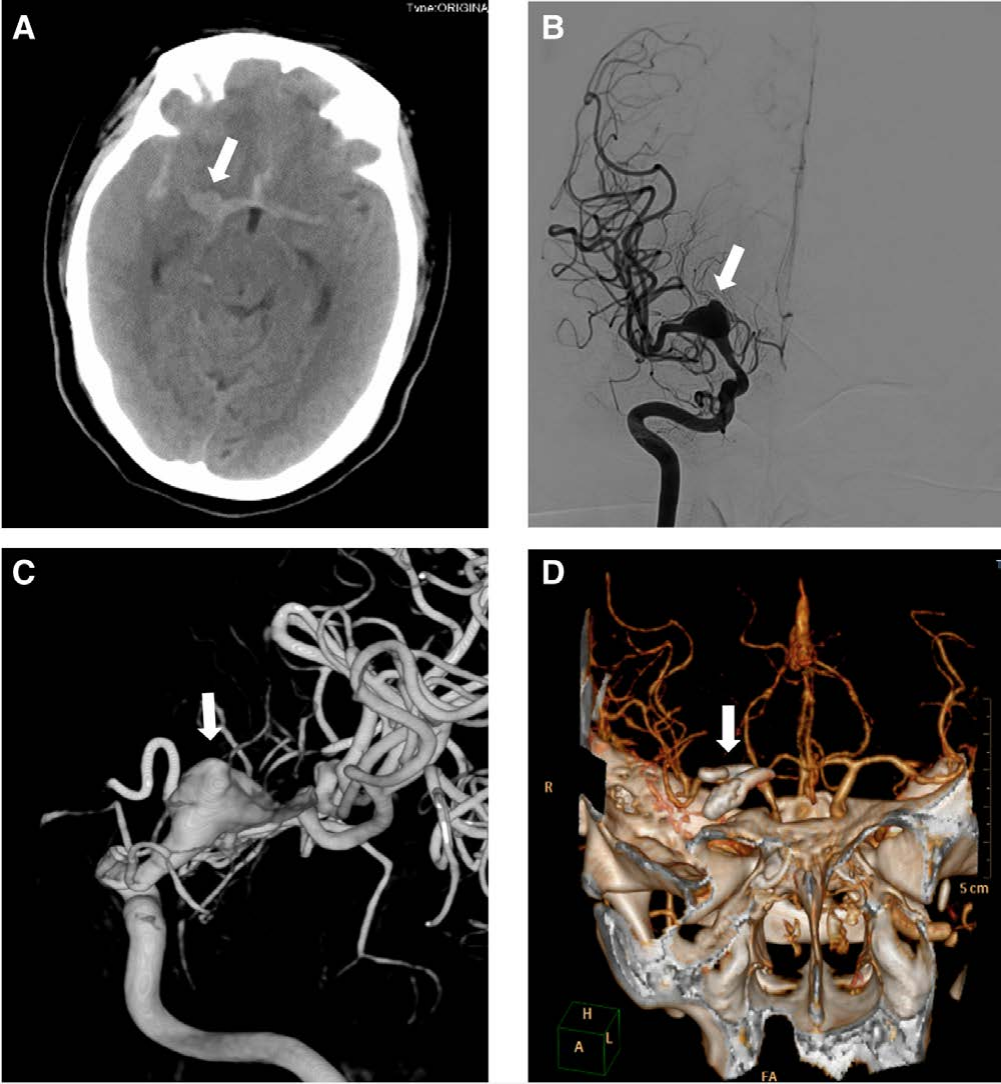
Neurosurgical clipping for the treatment of a ruptured aneurysm in the right middle cerebral artery (R-MCA) (A) Non-contrast CT scan showing subarachnoid hemorrhage and suspected R-MCA aneurysm. (B–C) Digital subtraction angiography images confirming recurrent of large spindle-shaped aneurysm in R-MCA. (D) Postoperative CTA examination showing complete clipping of R-MCA aneurysm. CT = computed tomography.

**Figure 3.**
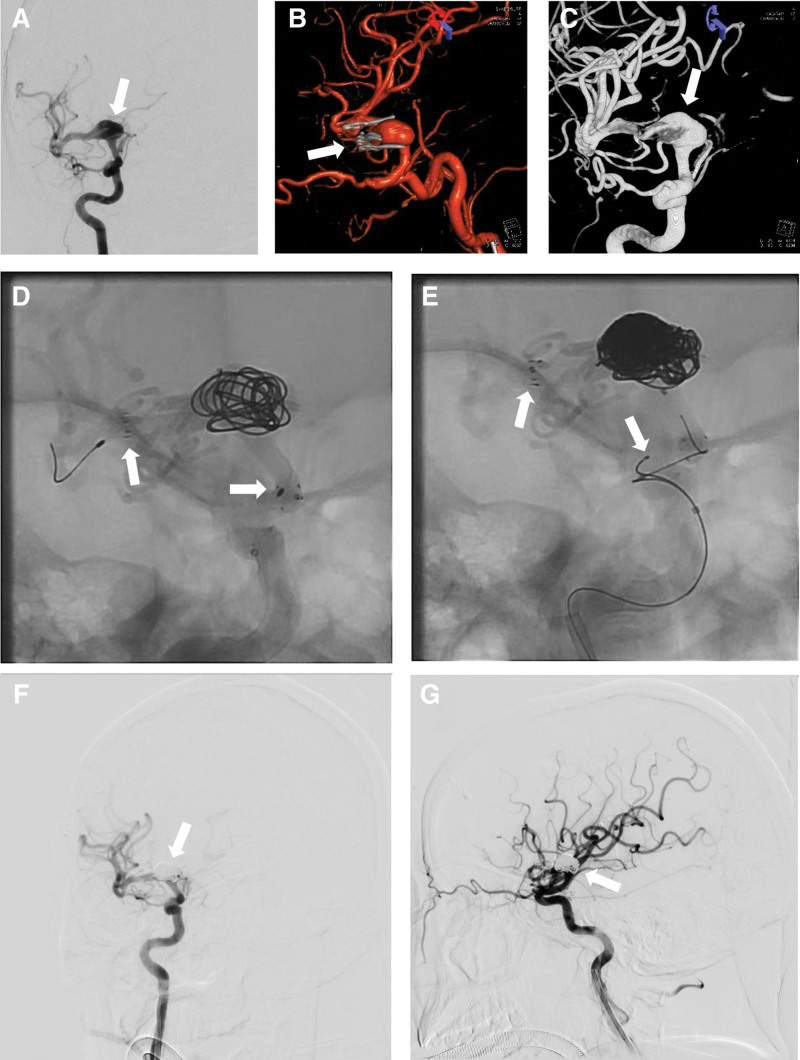
Stent-assist coil embolization treatment for recurrent middle cerebral artery aneurysm: (A–B) Angiographic image displaying the recurrent middle cerebral artery aneurysm and its corresponding aneurysm clip. (C) Remove the aneurysm clip and display the complete aneurysm. (D) A Neuroform EZ stent (4 mm × 30 mm) was implanted to assist in embolization of the aneurysm. (E) Implantation of a Solitaire AB Stent (6 mm × 30 mm) following continuous coil placement. (F–G) angiographic Images of recurrent aneurysms after stent-assisted embolization: anteroposterior angiographic image (F) and lateral angiographic image (G) confirming complete occlusion of the recurrent aneurysms and unobstructed middle cerebral artery.

In the third day after MT, we obtained images from 2 hospitals where the patient had prior treatments. The first hospital provided images of neurosurgical clipping (Fig. 2), while the second provided images of stent-assisted aneurysm coil embolization (Fig. 3). The patient history included a subarachnoid hemorrhage 2 years ago, revealing a large middle cerebral artery aneurysm (Fig. 2A–C). A CT angiogram (CTA) on the third day post-aneurysm clipping showed no residual or recurrent aneurysm (Fig. 2D). However, 18 months later, a recurrence was detected (Fig. 3A–C). Two stents, namely, 4 mm × 30 mm Neuroform EZ (Stryker) and 6 mm × 30 mm Solitaire AB (Medtronic, Irvine, California, USA), with the latter placed within the former, were used for endovascular coil embolization (Fig. 3D–E), resulting in complete embolization of the recurrent aneurysm (Fig. 3F and G). Subsequently, the patient recovery was uneventful, and she received aspirin (100 mg/day) and clopidogrel (75 mg/day) for secondary prevention of thromboembolism until her transfer to our hospital.

The patient ambulatory electrocardiogram (ECG) and cardiac ultrasound showed no signs of atrial fibrillation or abnormalities. The patient declined aspirin verify now and clopidogrel resistance testing. She was discharged from our hospital on the eighth day with focal neurological deficits (mRS 2/National Institutes of Health Stroke Scale score 5) and dual antiplatelet therapy, combining ticagrelor and aspirin, to reduce the risk of recurrent atherothrombotic events. Ticagrelor was discontinued 3 months after MT. In the 6-month follow-up, the patient mRS score was 1, and she remained free from recurrent cerebrovascular events.

## 3. Discussion

This case illuminates a complex clinical scenario involving acute ischemic stroke treated with thrombectomy. The patient history included neurosurgical clipping for an aneurysm, followed by the successful management of aneurysmal recurrence through stent-assisted coiling. Aneurysm recurrence post-microsurgical clipping, though rare, represents a formidable challenge due to the inability to achieve complete occlusion in all cases. Despite confirmed occlusion post-clipping, patients are exposed to the ongoing risk of recurrent aneurysms, carrying a heightened threat of rebleeding and mortality. The estimated incidence of aneurysm recurrence after microsurgical clipping stands at approximately 1% to 2%.^[8]^ The precise etiology of aneurysm recurrence remains incompletely understood, with factors such as incomplete occlusion, formation of new aneurysms at the same site, radiographically undetectable clip issues, or aneurysm regrowth being implicated.^[9]^

In our case, computed tomography angiography (CTA) conducted 2 days post-clipping demonstrated complete occlusion. Nevertheless, the risk of recurrence is influenced by diverse factors, including age, initial aneurysm size, and lifestyle. Notably, this case exhibited known risk factors for recurrence after clipping, including obesity, young age, and a large initial intracranial aneurysm size.^[10]^ Understanding these risk factors is crucial in comprehending the nuanced outcomes and challenges associated with the recurrence of intracranial aneurysms post-neurosurgical intervention.

Management of recurrent intracranial aneurysms after clipping typically involves endovascular and surgical techniques.^[11]^ Endovascular techniques, including embolization, coiling, stent-assisted coiling, and flow diverter devices, are less invasive than surgical techniques and are employed to treat aneurysms that are not amenable to surgical treatment.^[12]^ Surgical techniques such as clipping, trapping, and bypassing are reserved for recurrent intracranial aneurysms after clipping.^[13]^ In some cases, a combination of medical, endovascular, and surgical approaches may be used to manage recurrent intracranial aneurysms after clipping, with the specific treatment modality selected based on the aneurysm size, location, and morphology. In our case, the patient opted for stent-assisted coil embolization for managing recurrent cerebral aneurysms.

Endovascular coiling is a commonly used treatment approach for recurrent intracranial aneurysms, but it carries a low risk of thromboembolic events as a complication. Thromboembolic events are severe complications associated with embolization therapy for cerebral aneurysms, with morbidity rates of up to 45% and mortality rates of up to 23%.^[6]^ The exact mechanism underlying thromboembolic events after endovascular coiling is not fully understood, it is believed that disruption of the aneurysm wall plays a role. This disruption can lead to turbulent blood flow and thrombus formation, and the rough surfaces of the endovascular coils themselves can contribute to thrombus formation.^[14]^ Most thromboembolic events occur during the endovascular coiling procedure for intracranial aneurysms, whereas delayed thromboembolic events are rare, with a reported rate of 2.4% in patients with unruptured aneurysms.^[15,16]^ For acute embolic strokes, thrombolysis is a common treatment option, but it is contraindicated in cases with prior subarachnoid hemorrhage from ruptured aneurysms. Other strategies include pharmacotherapy, intra-arterial thrombolysis, and MT using techniques such as suction aspiration or stent retrievers. Intracranial stents are typically reserved as a last resort for salvage treatment.^[17]^

### 3.1. Limitations

Our study had several limitations. Firstly, we were unable to employ a suction and retrieval method with minimal vascular interference due to the inability to advance the suction catheter to the occlusion site in the middle cerebral artery. This limitation could be attributed to the presence of aneurysm clips occluding the middle cerebral artery and the use of 2 stents to assist with the coiling of a recurrent aneurysm. While the balloon-assisted tracking technique has been reported to facilitate passage through tortuous arterial segments,^[18]^ our center did not have a PTA balloon with a pusher long enough to perform this technique. In consideration of the possibility of the retrieval device entangling with the stent or coil used to treat the aneurysm, we opted for the use of a retrievable stent. Surgical removal of the thrombus causing occlusion of the middle cerebral artery remains an option, and if recurrent aneurysms are identified, surgery may be performed for both the recurrent aneurysm and the occluded thrombus.^[19,20]^ However, our center currently lacks the necessary capabilities to perform such procedures.

## 4. Conclusion

In conclusion, our case highlights the importance of regular follow-up in patients treated for intracranial aneurysms, particularly those with giant aneurysms. Our findings suggest that utilizing a retrievable stent for MT may be a viable therapeutic approach for extreme cases of delayed cerebral vascular occlusion following stent-assisted coil embolization in recurrent aneurysmal. Urgent clinical trials are warranted to evaluate the effectiveness of specialized thrombectomy devices tailored specifically for managing thromboembolic events arising after microsurgical clipping or coiling of cerebral aneurysms.

## Author contributions

**Conceptualization:** Ya Shao, Jinglun Li, Yushan Jiang, Zerui Xiang, Xue Zeng, Zhengzhou Yuan.

**Data curation:** Yuan Yang, Ting Wang, Jinglun Li, Yushan Jiang, Ziwei Yuan, Jiayi Tang, Zhou Yu.

**Formal analysis:** Ziwei Yuan.

**Investigation:** Yuan Yang, Ting Wang, Zhou Yu.

**Methodology:** Ya Shao, Ting Wang, Jinglun Li, Dihu Wang, Zhou Yu.

**Resources:** Dihu Wang.

**Supervision:** Zhongchun He.

**Validation:** Haidong Huang, Jiayi Tang, Zerui Xiang, Xue Zeng, Zhongchun He

**Visualization:** Yushan Jiang, Xue Zeng, Zhongchun He.

**Writing – original draft:** Ya Shao, Yuan Yang, Haidong Huang, Ziwei Yuan, Xue Zeng, Zhengzhou Yuan.

**Writing – review & editing:** Zhengzhou Yuan.
